# Prefrontal cortex stimulation normalizes deficient adaptive learning from outcome contingencies in low mood

**DOI:** 10.1038/s41398-024-03204-3

**Published:** 2024-12-18

**Authors:** Verena Sarrazin, Margot Juliëtte Overman, Luca Mezossy-Dona, Michael Browning, Jacinta O’Shea

**Affiliations:** 1 Wellcome Centre for Integrative Neuroimaging, University of Oxford, OX3 9DU Oxford, United Kingdom; 2 Department of Psychiatry, Warneford Hospital, University of Oxford, OX3 7JX Oxford, United Kingdom; 3 Oxford Centre for Human Brain Activity (OHBA), University of Oxford, OX3 7JX Oxford, United Kingdom

**Keywords:** Depression, Learning and memory

## Abstract

Depression and anxiety are associated with deficits in adjusting learning behaviour to changing outcome contingencies. This is likely to drive and maintain symptoms, for instance, by perpetuating negative biases or a sense of uncontrollability. Normalising such deficits in adaptive learning might therefore be a novel treatment target for affective disorders. The aim of this experimental medicine study was to test whether prefrontal cortex transcranial direct current stimulation (tDCS) could normalise these aberrant learning processes in depressed mood. To test proof-of-concept, we combined tDCS with a decision-making paradigm that manipulates the volatility of reward and punishment associations. 85 participants with low mood received tDCS during (or before) the task. In two sessions participants received real or sham tDCS in counter-balanced order. Compared to healthy controls (*n* = 40), individuals with low mood showed significantly impaired adjustment of learning rates to the volatility of loss outcomes. Prefrontal tDCS applied during task performance normalised this deficit, by increasing the adjustment of loss learning rates. As predicted, prefrontal tDCS before task performance (control) had no effect. Thus, the effect was cognitive-state dependent. Our study shows, for the first time, that a candidate depression treatment, prefrontal tDCS, when paired with a task, can reverse deficits in adaptive learning from outcome contingencies in low mood. Thus, combining neurostimulation with a concurrent cognitive manipulation is a potential novel strategy to enhance the effect of tDCS in depression treatment.

## Introduction

Transcranial direct current stimulation (tDCS), a non-invasive brain stimulation method, applies constant electric currents through the scalp to change cortical excitability. TDCS is under investigation as an antidepressant treatment. Depression is associated with hypoactivity in the left dorsolateral prefrontal cortex (DLPFC) [1, 2]. In depression trials, a bifrontal tDCS montage is commonly used, applying anodal (excitatory) tDCS to left, and cathodal (inhibitory) tDCS to right DLPFC [3, 4]. A recent meta-analysis suggests that tDCS applied to DLPFC has mild-to-moderate antidepressant effects [5]. More research is needed to understand the mechanisms of tDCS action and improve efficacy.

Depression is typically characterised by a negative cognitive bias, i.e. information processing is biased towards negative rather than positive information [2]. Compared to healthy controls, individuals with depressive symptoms remember more negative words [6], perceive feedback as more negative [7] and tend to interpret ambiguous information as negative [8]. Negative biases are hypothesised to play a causal role in the development and maintenance of depressive symptoms [2, 9, 10]. Reduction in negative bias has been shown to be one mechanism of action of antidepressant drugs [9–11]. Preliminary evidence indicates that bifrontal tDCS might also have the potential to reduce negative biases in depression or anxiety [12, 13].

Recent research in computational psychiatry has shed light on how negative biases might develop. Information processing should prioritise learning from outcomes that are most informative, i.e. most useful for predicting future outcomes [14]. Informativeness depends in part on the volatility of the underlying reward association [14, 15]. If the association is volatile (i.e. changes over time) compared to stable, an unexpected outcome is more likely to signal a change in the underlying reward association, i.e. it is more informative. In a volatile environment, behaviour should therefore be changed more quickly in response to unexpected outcomes than in stable environments, i.e. learning rates should be higher in volatile environments [14, 15]. Learning rates can therefore be interpreted as a measure of the estimated informativeness of outcomes.

Healthy individuals adjust their learning rates to volatility [14, 16]. Anxiety and depression have been associated with deficits in learning rate adjustment, i.e. with estimating the informativeness of outcomes and adjusting behaviour accordingly [17, 18]. Such deficits could lead to a negative bias if, for example, individuals estimated negative events to be more informative than positive events [19, 20], and hence disproportionally focused their attention on negative outcomes. On a computational level, this could manifest as increased punishment vs. reward learning rates [21–23], which could lead to maladaptive behaviour, e.g. causing an individual to give up quickly after negative feedback, preventing possible future positive outcomes. Potential cognitive treatment targets could therefore be to normalize deficits in learning rate adjustment, and/or negative bias in punishment vs. reward learning rates.

The aim of this study was to investigate whether bifrontal tDCS could normalize reinforcement learning deficits in low mood. DLPFC is part of a brain network involved in reinforcement learning and is activated in response to volatility [24–26]. In our previous study, we found that bifrontal tDCS increased reward learning rates in healthy volunteers [27]. However, given changes in reinforcement learning, it was unclear whether the same tDCS effect should be expected in low mood. To assess this, here we compared task performance between healthy volunteers from our previous study and individuals with depressive symptoms [27]. We hypothesised that individuals with depressive symptoms would show reduced adjustment of learning rates to volatility [17, 18] and/or increased punishment vs. reward learning rates [21–23]. We then tested whether bifrontal tDCS applied during task performance could normalise these expected learning deficits in low mood.

Our secondary aim was to test the hypothesis that tDCS would have a greater functional impact when applied during rather than before task performance. When applied ‘online’, i.e. during activity-dependent neuroplasticity, tDCS has been shown to change learning, with no such effect when applied ‘offline’ during rest prior to plasticity induction [27–30]. We therefore hypothesised that tDCS would normalise deficits in reinforcement learning only when applied *during* but not *before* task performance.

## Methods

This study has been pre-registered (https://clinicaltrials.gov/ct2/show/NCT03393312). Analyses not included in our pre-registration are marked as ‘not pre-registered’. A justification for these deviations is provided in the ‘Deviations from pre-registration’ section. Data and analysis scripts are available on Open Science Framework (10.17605/OSF.IO/KJB6Y).

### Sample

85 community volunteers suffering from low mood (Beck Depression Inventory II [31] score of at least 10) were recruited via university email lists and social media advertisements (see Table 1 for demographic details). Participants were excluded from the study if they had any contraindication to tDCS, such as medication (apart from the contraceptive pill), neurological conditions, a family history of epilepsy, metal implants inside the brain, or current pregnancy. 41 participants were assigned to the “tDCS during task” group, and 44 participants to the “tDCS before task” group. All participants completed two testing sessions in which they received real or sham tDCS in counter-balanced order. An a-priori power analysis based on the tDCS effect size from our previous study in healthy participants [27] indicated that a minimum sample size of 38 participants per group was required to achieve 80% power for contrasting the effect of real vs. sham tDCS (paired t-test, two-tailed, *Cohen’s dz* = 0.472). This study was approved by the University of Oxford Central University Ethics Committee (R67041/RE002). All participants gave written informed consent to take part in the study.

**Table 1:** Mean (SD) baseline characteristics for the ‘general population’ and ‘low mood’ samples, and the “tDCS during task” and “tDCS before task” groups.

*Effect of low mood analysis*	General population (*n* = 40)	Low mood (*n* = 43)
*Sociodemographic data*		
Female (%)	25 (62%)	32 (74%)
Male (%)	15 (38%)	11 (26%)
Age in years (SD)	25.7 (5.5)	24.4 (4.6)
*Clinical measures*		
STAI-Trait	35.7 (7.2)	56.9 (8.9)
BDI	4.7 (6.5)	26.8 (9.7)

*Effect of tDCS analysis*	tDCS during task (*n* = 41)	tDCS before task (*n* = 44)
*Sociodemographic data*		
Female (%)	24 (59%)	30 (68%)
Male (%)	17 (41%)	14 (32%)
Age in years (SD)	24.3 (4.8)	24.2 (4.6)
*Clinical measures*		
STAI-Trait	55.4 (9.0)	57.4 (8.3)
BDI	24.9 (9.1)	27.7 (8.4)

*BDI* Beck Depression Inventory-II, score range = 0–63, *STAI-Trait* State-Trait Anxiety Inventory (trait form), score range = 20–80.

To investigate the effect of low mood on learning behaviour, we first compared task performance at baseline (i.e. during sham tDCS) between the participants with low mood and the healthy participants from our previous study [27](not pre-registered). To avoid confounds from task repetition, only individuals (from either study) who received sham tDCS in their first session were included in this analysis (low mood: *n* = 43, healthy: *n* = 40). Demographic data and baseline questionnaire scores for both samples are shown in Table 1.

### Information bias learning task

**Fig. 1:**
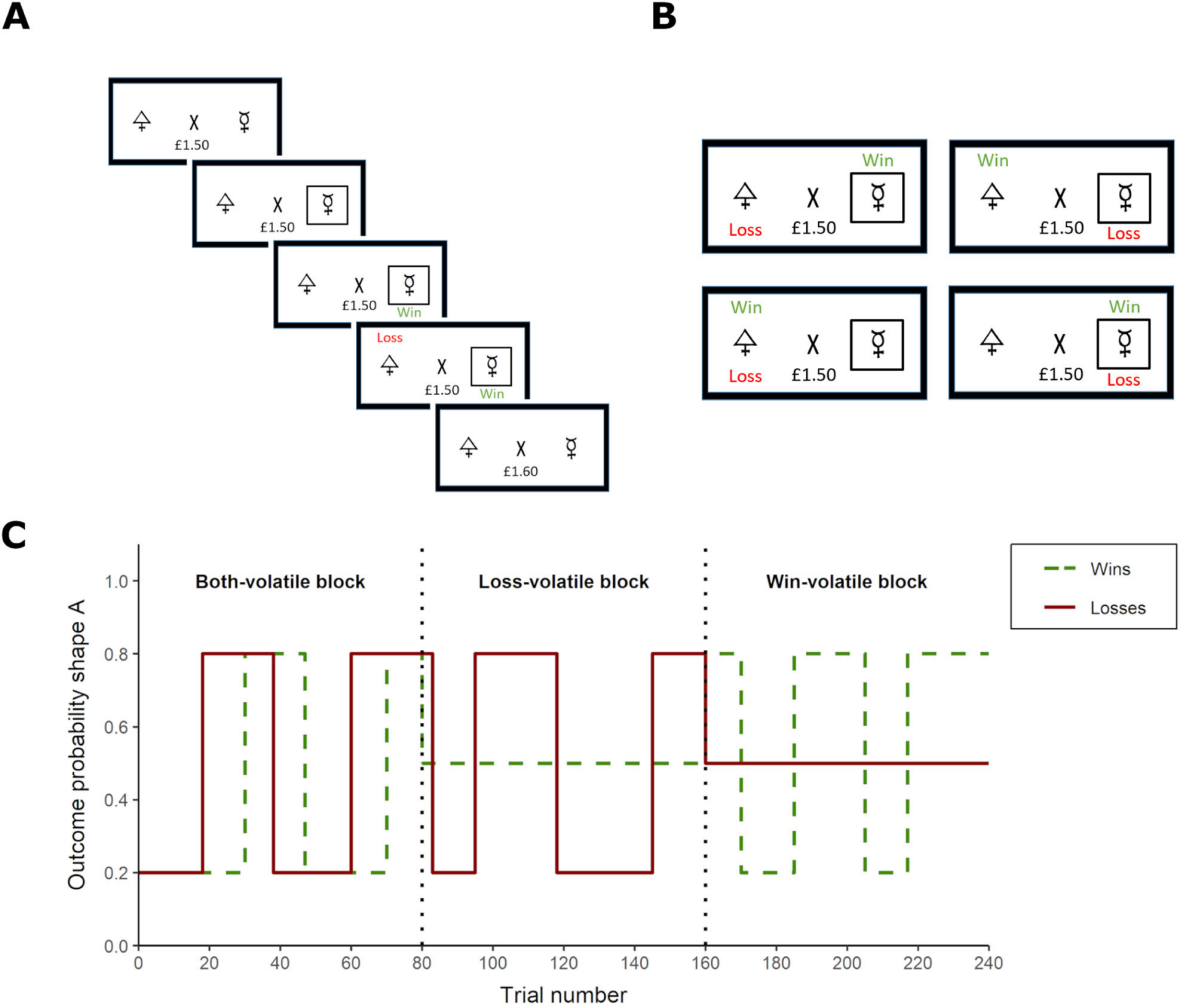
Task design of the information bias learning task. **A** On each trial, participants were asked to choose one of two shapes, by pressing the keys ‘A’ or ‘L’ for the left or right shape, respectively. Subsequently, a win and a loss outcome appeared on the screen. **B** The win and loss outcomes were independent of each other, resulting in four possible scenarios: The chosen shape might be associated with the win (+10p), the loss (−10p), both outcomes (0p) or neither (0p). Wins and losses were associated with an actual win or loss of 10p on each trial, respectively. (**C**) Underlying reward structure for a ‘both-volatile’, ‘losses-volatile’ and ‘wins-volatile’ block. In this task, the volatility of the wins and losses was manipulated independently. In ‘wins-volatile’ blocks, the wins were associated with one of the shapes in 80% of the trials, and with the other in 20%. This association reversed a few times within the block. Losses were randomly presented with either shape (50%) and are therefore uninformative. In ‘losses-volatile’ blocks, the probability pattern was reversed. In ‘both-volatile’ blocks, both wins and losses were independently associated with one shape in 80% and with the other in 20. Reproduced with permission from ref. [27].

The Information Bias Learning Task [19] manipulates the relative informativeness of win and loss outcomes. The task is described in detail in Fig. 1. Briefly, participants performed six blocks of 80 trials of choosing between two shapes. Each trial resulted in a win (+10p), a loss (−10p), both win and loss (0p), or neither outcome (0p). Wins and losses were independently associated with the two shapes which allowed for separate estimation of win and loss learning rates. The relative informativeness of wins and losses was manipulated throughout the six blocks, such that wins and losses were equally informative (both-volatile), wins were more informative than losses (wins-volatile) or losses were more informative than wins (losses-volatile).

### tDCS protocol

**Fig. 2:**
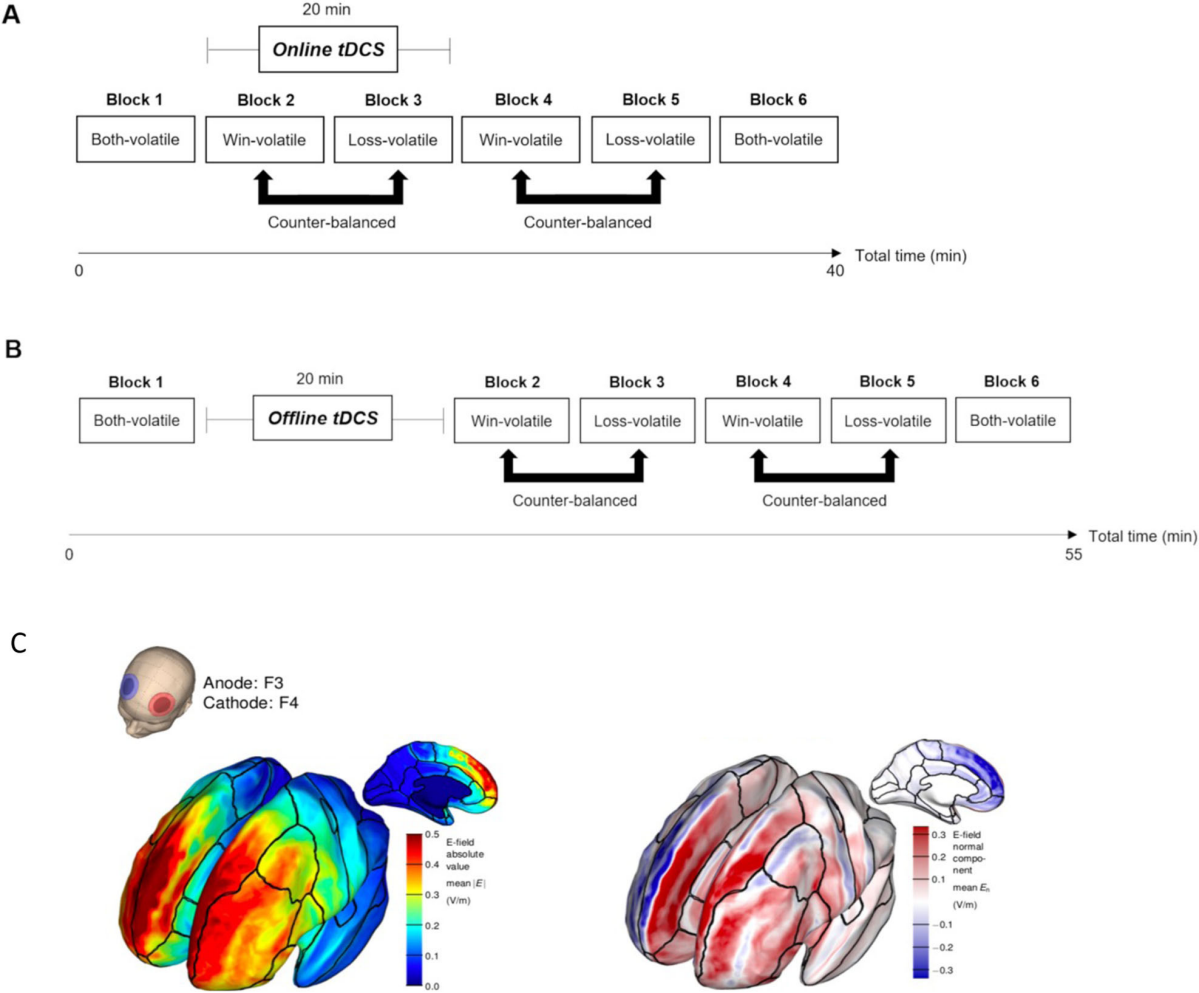
Study design. **A** Task protocol for the “tDCS during task” group. Participants started with a ‘both-volatile’ block, and then underwent two ‘wins-volatile’ and two ‘losses-volatile’ blocks in alternating order. Half of the participants performed the ‘wins-volatile’ block first, while the other half performed the ‘losses-volatile’ block first. The experiment ended with another ‘both-volatile block’. Stimulation was applied during the performance of block 2 and 3. **B** Task protocol for the “tDCS before task” group. The task protocol was identical to **A** with the exception that tDCS was applied at rest after performance of the first task block. **C** Modelling of the electric field induced by the bifrontal tDCS setup, with the anode over left, and the cathode over right DLPFC. The left figure shows the strength of the electric field. The right figure displays the normal component (red = anodal stimulation, blue = cathodal stimulation). Adapted from ref. [27] with permission. **C** is adapted from ref. [40] with permission.

All participants took part in two testing sessions (minimum 1 week interval) where they received real or sham tDCS in counter-balanced order (double-blinded). Real tDCS was applied for 20 minutes at an intensity of 2mA. The anode and cathode were placed over left and right DLPFC, respectively, approximated by the F3 and F4 electrode positions (international 10–20 system)(see Fig. 2C for a simulation of the electric field). All participants performed the first task block (“both-volatile”) without tDCS. Then the ‘online’ group received tDCS while performing the second and third task blocks (“tDCS during task” group, Fig. 2A) and completed blocks 4–6 after tDCS had ended. The ‘offline’ group received tDCS at rest immediately after the first task block. They then performed blocks 2–6 immediately after the stimulation period (“tDCS before task” group, Fig. 2B).

### Computational modelling

Performance in the Information Bias Learning Task was analysed using computational models that were fitted to participants’ trial-by-trial choices. The fit of six models was compared using the Bayesian Information Criterion (BIC) averaged across participants (see Supplementary S1). All models estimated win and loss probabilities which were updated using learning rate parameters and transformed into choice probabilities using a Softmax function including inverse temperature parameters capturing choice stochasticity. We tested two modelling approaches: “block-wise” and “constant”. In the “block-wise models”, learning rate and inverse temperature parameters were fitted separately to each task block, as in previous work in healthy volunteers [19]. However, since the inverse temperature in that study did not vary between task blocks we also tested a simpler approach, in which the same inverse temperature parameter was fitted across all six task blocks (“constant model”)(not pre-registered). The “constant model” approach captured predicted differences between healthy volunteers and low mood, whereas the “block-wise” approach did not. Hence, all results reported here are based on the “constant model”. Results from the “block-wise” model are reported in the Supplementary Material S3.2, S4.3.

Model comparison was performed between six comparator models using the BIC averaged across participants. Statistical analysis was performed based on the parameter estimates derived from the winning model. The winning model used a modified version of a Rescorla-Wagner updating rule in which the probability of an outcome being associated with shape A was modelled separately for win and loss outcomes:

$${{rwin}}_{\left(i+1\right)}={{rwin}}_{(i)}+\alpha {win}* ({{winout}}_{(i)}-{{rwin}}_{(i)})$$

$${{rloss}}_{\left(i+1\right)}={{rloss}}_{(i)}+\alpha {loss}* ({{lossout}}_{(i)}-{{rloss}}_{(i)})$$

Where ${{rwin}}_{\left(i+1\right)}$ and ${{rloss}}_{\left(i+1\right)}$ are the estimated probabilities of the win or loss being associated with shape A on trial *i* + *1*. These probability estimates were updated on each trial with the prediction error on the previous trial weighted by the win or loss learning rate, $\alpha {win}$ or $\alpha {loss}$. A Softmax function was used to transform the probability estimates into choice probabilities:

$${P}_{\left({choice}=A\left(i\right)\right)}=\frac{1}{1+{\exp }^{\left(-\left(\beta {win}* \left({rwi}{n}_{\left(i\right)}-0.5\right)-\beta {loss}* \left({rlos}{s}_{\left(i\right)}-0.5\right)\right)\right)}}$$

Where *P(choice* = *A)*_*(i)*_ represents the probability of the participant choosing shape A on trial *i*. The model contained two inverse temperature parameters, $\beta {win}$ and $\beta {loss}$ which capture sensitivity to win and loss outcomes, respectively. A larger inverse temperature estimate indicates that the estimated probability of the respective outcome was taken more into account, whereas smaller estimates indicate more random choice behaviour. The two inverse temperature parameters were estimated across all six blocks.

Parameters for the constant models were estimated in STAN [32] (see Supplementary S1.2). An inverse logit transformation was applied to the learning rate estimates. The inverse temperature estimates were log-transformed.

### Statistical analysis

#### Computational modelling outcome variables

The main outcome measures of interest were win and loss learning rates, and their relative adjustment between volatile versus stable blocks, defined as follows:

*win learning rate adjustment* = win learning rate _wins-volatile condition_ – win learning rate _losses-volatile condition_

*loss learning rate adjustment* = loss learning rate _losses-volatile condition_ – loss learning rate _wins-volatile condition_

*learning rate adjustment bias* = loss learning rate adjustment – win learning rate adjustment

Positive learning rate adjustment values indicate that learning rates were higher in volatile than in stable conditions. The *learning rate adjustment bias* captures the extent to which learning rate adjustment was biased towards either win or loss outcomes. A positive value on *learning rate adjustment bias* therefore indicates that loss learning rates were adjusted more to changes in informativeness than win learning rates.

#### Non-computational outcome variables

To ensure that observed effects did not depend on specific computational modelling choices, we also conducted non-computational analyses to cross-validate the key findings. Logistic regressions were run to predict the choice on each trial using win and loss outcomes of the previous 3 trials as regressors:

$${\rm{Choice}}({\rm{n}}) \sim {\rm{win}}({\rm{n}}-1)+{\rm{loss}}({\rm{n}}-1)+{\rm{win}}({\rm{n}}-2)+{\rm{loss}}({\rm{n}}-2)+{\rm{win}}({\rm{n}}-3)+{\rm{loss}}({\rm{n}}-3)$$

This is explained in more detail in Supplementary S5.

All analyses were performed in RStudio (Version 1.4.1717, R 4.1.1). Outcome measures were analysed in repeated-measures ANOVAs (*ezANOVA* package). The two main factors of interest were: Sample (low mood vs. healthy volunteers) or tDCS Condition (real vs. sham) and their hypothesized interactions with within-subjects factors of Valence (win vs. loss) and Volatility (both-volatile, wins-volatile and losses-volatile). Additional factors were: Block Order (wins-volatile first vs. losses-volatile first; between-subjects factor of no interest) and Time (first half vs. second half of task) was included in the analysis of the effect of low mood to account for the repeated task conditions (each of the three Volatility conditions (both-volatile, wins-volatile, losses-volatile) was performed twice per session, see Fig. 2). Analyses of the effect of tDCS focused on blocks 2 and 3 (i.e. the two blocks during stimulation for the “tDCS during task” group and immediately after stimulation for the “tDCS before task” group). Analyses were designed to test the following hypotheses:

#### Effect of low mood

H1(a): Individuals with low mood will show increased loss vs. win learning rates compared to healthy volunteers.

H1(b): Individuals with low mood will show decreased adjustment of learning rates to volatility compared to healthy volunteers.

#### Effect of tDCS

H2: TDCS *during* task performance will normalise the deficits observed in low mood.

H3: TDCS *before* task performance will not induce the same effects (as in H2).

Table 2 states which outcome measures and factors were included in each analysis. Summary statistics for all computational parameters are provided in Supplementary S6.

**Table 2:** Outcome measures and factors included in the repeated-measures ANOVAs for each hypothesis.

Hypothesis	Outcome measure	ANOVA factors
*Effect of low mood*		
H1(a): Individuals with low mood will show increased loss vs. win learning rates compared to healthy volunteers.	Learning rates	Sample (low mood vs. healthy volunteers) Valence (win vs. loss) Volatility (both-volatile, wins-volatile and losses-volatile) Time (first half vs. second half) Block Order (wins-volatile first vs. losses-volatile first)
H1(b): Individuals with low mood will show decreased adjustment of learning rates to volatility compared to healthy volunteers.	Learning rate adjustment	Sample Valence Time Block Order
	Learning rate adjustment bias	Sample Time Block Order
*Effect of tDCS* These analyses focus on blocks 2 and 3 (i.e. during stimulation for “tDCS *during* task” group vs immediately after for “tDCS *before* task” group).		
H2: TDCS *during* task performance will normalise the reinforcement learning deficits observed in low mood.	Learning rates	tDCS Condition (real vs. sham) Valence Volatility (wins-volatile vs. losses-volatile) Block Order
	Learning rate adjustment	tDCS Condition Valence Block Order
	Learning rate adjustment bias	tDCS Condition Block Order
H3: TDCS applied *before* task performance will not have the same effect as tDCS applied *during* task performance.	Learning rates Learning rate adjustment Learning rate adjustment bias	Identical to H2 plus t-test to contrast the effect of tDCS *before* vs. tDCS *during* task performance

All outcome measures were tested for correlations with BDI and trait anxiety scores. Significance of correlations was assessed using t-tests.

### Outlier handling

All analyses were repeated after removing outliers (not pre-registered). A datapoint was identified as an outlier if it was more than 1.5 times the interquartile range below the first or above the third quartile. For each outcome measure, outliers were removed separately for the levels of the factors of interest (i.e. separately for the healthy volunteer vs. low mood groups, and win vs. loss outcomes) to ensure that the data distribution for each factor level was not excessively biased by potential outliers (caused by inattention, misunderstanding of instructions etc.). For the analysis of the effect of tDCS, a datapoint was identified as an outlier if the difference between real minus sham tDCS was more than 1.5 times the interquartile range below the first or above the third quartile. This method was chosen to ensure that the estimated effect of tDCS was not biased by participants showing an unrepresentative effect size. Statistics are reported for the entire dataset (i.e. without outlier removal) unless outlier removal had an impact on the results, in which case statistics are reported both with and without outlier removal (for analyses that outlier removal had an impact on, figures including outliers are included in the Supplementary Material). For the analysis of the effect of tDCS, an additional non-parametric Wilcoxon signed-rank test was conducted on all data points, as a more robust analysis which is less sensitive to outliers.

## Results

### Low mood is associated with biased learning rate adjustment

**Fig. 3:**
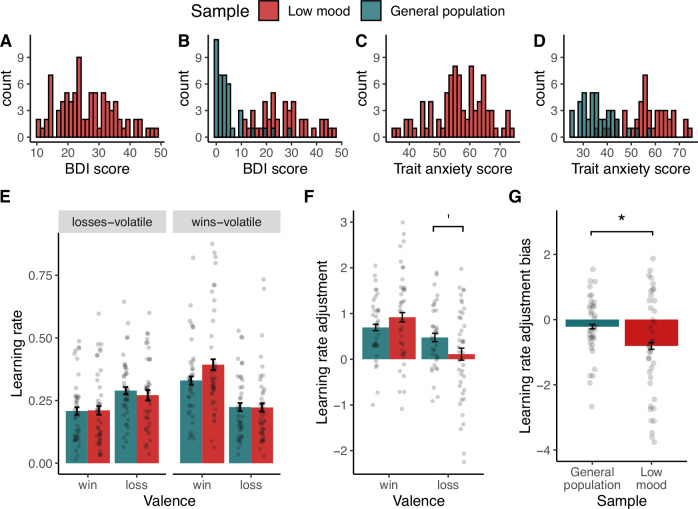
Distribution of BDI and STAI trait anxiety scores (top row) and effect of low mood on learning rate measures (bottom row). **A** and **C** show the distribution of BDI and trait anxiety scores of all participants recruited for this study (*n* = 85). **B** and **D** show the distributions of BDI and trait anxiety scores for participants included in the comparison between the low mood sample (*n* = 43) and general population sample (*n* = 40). Participants with low mood had significantly higher BDI and trait anxiety scores than participants in the general population sample (two-sample Welch t-test: BDI: *t*(73.8) = 12, *p* < 0.001; STAI-T: t(79) = 11.7, *p* < 0.001)). **E** Low mood did not affect learning rates per se, in contrast with H1(a) and previous reports of increased punishment learning rates in depression [21–23]. **F** Confirming H1(b) there was a significant interaction effect between Sample and Valence on learning rate adjustment. Participants with low mood showed a trend towards lower loss learning rate adjustment. **G** Reflecting the significant interaction effect in **F**, participants with low mood showed a significant learning rate adjustment bias, i.e. significantly lower adjustment of loss relative to win learning rates. Overall, these findings confirm H1(b), showing impaired learning rate adjustment in low mood. Specifically, loss learning rates were adjusted less than win learning rates. Asterisk * indicates *p* < 0.05; ‘ indicates a trend. Error bars indicate standard error of the mean (SEM).

Contrary to H1(a), there was no effect of Sample on learning rates, indicating that individuals with low mood did not show a significant increase in loss vs. win learning rates (no main effect of Sample (*p* = 0.46), no effect of Sample on win (*p* = 0.79) or loss learning rates (*p* = 0.12) (Fig. 3E, see Supplementary S3.3).

Confirming H1(b), there was a significant Sample x Valence interaction on *learning rate adjustment* (*F*(1,72) = 4.4, *p* = .038, seven outliers removed; before outlier removal: *F*(1,79) = 4.0, *p* = 0.046; Fig. 3F and Supplementary Fig. S11). Post-hoc tests indicated no significant main effect of Sample on win learning rate adjustment (*F*(1,72) = 1.3, *p* = 0.24) but a trend towards lower loss learning rate adjustment in the sample with low mood (*F*(1,72) = 3.2, *p* = 0.076; Fig. 3F). Reflecting the significant interaction effect, individuals with low mood showed a significant *learning rate adjustment bias* (main effect of Sample: *F*(1,72) = 4.4, *p* = 0.038, *Cohen’s d* = 0.44, same seven outliers removed; before outlier removal: *F*(1,79) = 4.0, *p* = 0.046; Fig. 3G). That is, while the general population adjusted their win and loss learning rates to a similar extent (*learning rate adjustment bias* not different from zero (one-sample t-test): *t*(35) = −1.3, *p* = 0.17), individuals with low mood adjusted their loss learning rate significantly less than their win learning rate (*learning rate adjustment bias* significantly below zero: *t*(39) = −3.2, *p* = 0.002). Accordingly, there was a negative correlation between BDI score and *learning rate adjustment bias* across groups (r = −0.25, *t*(75) = −2.2, *p* = 0.027 although correlations within groups were non-significant, see Supplementary Fig. S12).

### Online bifrontal tDCS normalizes learning rate adjustment in low mood

**Fig. 4:**
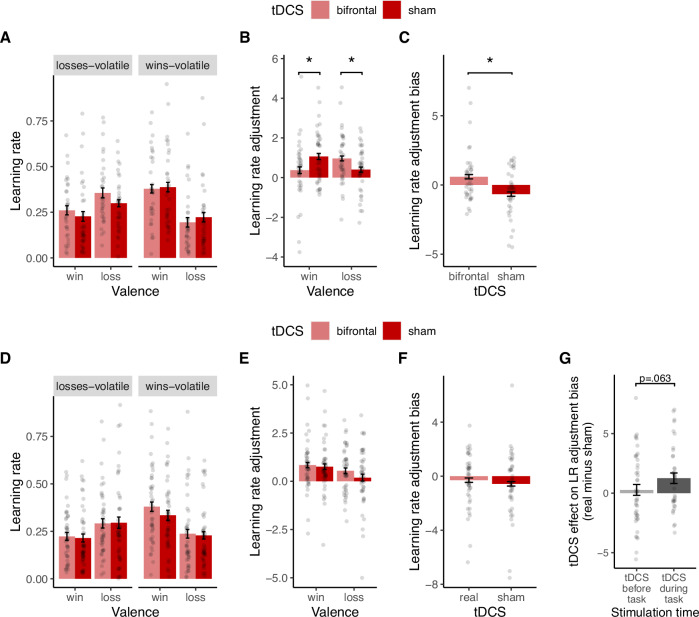
Effect of bifrontal tDCS applied *during* task performance (top row) and effect of tDCS applied *before* task performance (bottom row) on learning rate measures. Top row: **A** tDCS had no effect on learning rates per se. **B** Confirming H2, tDCS normalized the learning rate adjustment deficit in low mood. There was a significant interaction between tDCS and Valence. Compared to sham, bifrontal tDCS *during* the task led to a significant increase in loss learning rate adjustment, and a significant decrease in win learning rate adjustment. **C** Reflecting the significant interaction effect in **B**, the significant negative *learning rate adjustment bias* in the sham condition was abolished by tDCS. Overall, these findings confirm H2, showing that bifrontal tDCS during the task normalized impaired learning rate adjustment in low mood. Bottom row: No effect of bifrontal tDCS applied *before* task performance on learning rates. Real compared to sham tDCS applied during task performance did not have any significant effect on learning rates (**D**), learning rate adjustment (**E**) or learning rate adjustment bias (**F**). **G** Comparison of the effects of bifrontal tDCS applied *during* vs. *before* task performance on *learning rate adjustment bias*. Whereas stimulation applied *during* the task had a significant effect and abolished the *learning rate adjustment bias*, stimulation *before* the task had no effect. The direct statistical contrast of the two tDCS effects confirmed a trend (*p* = 0.063, one-tailed) towards a larger effect of tDCS applied *during* task performance. Asterisk * indicates *p* < 0.05. Error bars indicate SEM.

As outlined above, there was no deficit in learning rates per se in low mood. Accordingly, tDCS had no effect on learning rates per se (all *p* > 0.60, Fig. 4A, see Supplementary S4.2).

Learning rate adjustment was impaired in low mood (Fig. 3). Confirming H2, tDCS normalized this deficit. There was a significant tDCS Condition x Valence interaction on learning rate adjustment (*F*(1,36) = 7.9, *p* = .008, three outliers removed; before outlier removal: *F*(1,39) = 4.0, *p* = 0.052; Fig. 4B and Supplementary Fig. S13). Real compared to sham tDCS led to an increase in loss learning rate adjustment (*F*(1,36) = 6.1, *p* = 0.018, *Cohen’s dz* = 0.46), and a decrease in win learning rate adjustment (*F*(1,36) = 4.2, *p* = 0.047, *Cohen’s dz* = 0.48; Fig. 4B). Consistent with this, real vs. sham tDCS abolished the *learning rate adjustment bias* (main effect of tDCS: *F*(1,36) = 7.9, *p* = 0.008, *Cohen’s dz* = 0.65, same three outliers removed; before outlier removal: *F*(1,39) = 4.0, *p* = 0.052; Fig. 4C). A Wilcoxon signed-rank test (a non-parametric test less sensitive to outliers) including all data points confirmed a significant effect of tDCS on *learning rate adjustment bias* (*V* = 586, *p* = 0.044). During sham tDCS, *learning rate adjustment bias* was negative and differed significantly from zero (*t*(37) = 2.1, *p* = 0.037), indicating that participants adjusted their loss learning rates significantly less than their win learning rate. During real tDCS, *learning rate adjustment bias* did not differ significantly from zero (*t*(37) = 1.8, *p* = 0.067), indicating that tDCS abolished *learning rate adjustment bias*. To test whether the effect outlasted the stimulation period, an additional ANOVA was run on blocks 3 and 4 after the stimulation had ended. The effect on learning rate adjustment did not outlast the stimulation period (no significant effect of tDCS Condition in blocks 3 and 4, Supplementary Fig. S14).

### No effect of bifrontal tDCS applied before task performance

We further hypothesised (H3) that the effect of bifrontal tDCS would occur specifically when applied during task performance (“tDCS during task”) and not during rest (“tDCS before task”). As predicted, tDCS *before* task performance had no effect on learning rates or learning rate adjustment (all *p* > 0.30, see Supplementary S4.2) (Fig. 4D–F). Whereas tDCS applied *during* task performance abolished the *learning rate adjustment bias* (*p* = 0.008), tDCS applied *before* task performance had no such effect (*F*(1,42) = 0.06, *p* = 0.79; Fig. 4F). The direct statistical contrast between the two groups (tDCS during vs. before task) confirmed a trend towards a stronger effect of tDCS during task performance (*t*(78.9) = −1.5, *p* = 0.063 (Welch two sample t-test, one-sided)(Fig. 4G).

### Non-computational validation

To cross-validate the findings from the computational model, we ran logistic regressions including separate regressors for the outcomes of the three previous trials, and tested whether the two main findings (i.e. effect of low mood and tDCS on learning rate adjustment) related to the regression weights (see Supplementary Material S5). Regression weights for trial-by-trial outcomes capture similar behavioural characteristics to learning rates, without relying on a specific computational model. A higher learning rate corresponds to higher weight on the most recent outcomes. During real tDCS (during task), individuals with low mood adjusted the weight on the loss outcome from the previous trial more to informativeness than during sham tDCS (marginal effect of tDCS on loss learning rate adjustment: *p* = 0.069; Supplementary Fig. S21A). This is conceptually equivalent to the observed increase in loss learning rate adjustment. Regarding the effect of low mood on learning rate adjustment, the findings from the regression analysis were mixed (Supplementary Fig. S23).

## Discussion

The goal of this study was to investigate whether bifrontal tDCS could normalise deficits in adaptive learning from outcome contingencies in low mood. Participants with low mood performed a task that manipulated the relative informativeness (volatility) of positive and negative outcomes. Compared to healthy participants, individuals with low mood did not show a negative bias in learning rates, i.e. increased loss vs. win learning rates. However, low mood was associated with reduced adjustment of loss compared to win learning rates to changes in informativeness. Bifrontal tDCS applied during task performance normalised this learning rate adjustment bias, by increasing the adjustment of loss compared to win learning rates. This effect was cognitive-state-dependent, as it occurred only when stimulation was applied *during* task performance; bifrontal tDCS applied *before* task performance had no effect. However, these findings were limited to one of the two computational modelling approaches (see limitations below, and Supplementary Material for the results of the “block-wise” modelling approach).

Negative biases in depression are hypothesised to arise from aberrant reinforcement learning. Depressive symptoms have been associated with negatively biased learning rates, i.e. increased punishment vs. reward learning rates [21–23]. However, in contrast to our hypothesis (H1(a)), there was no evidence for increased loss vs. win learning rates in low mood in this study (see also refs. [33–35]).

Anxious-depressive symptoms have been associated with deficits in adjusting learning rates to the volatility of outcome contingencies [17, 18]. Consistent with this hypothesis (H1(b)), in this study, participants with low mood showed deficits in adjusting their learning rates to changes in informativeness. While healthy participants adjusted their win and loss learning rates to an equal extent, participants with low mood adjusted their loss learning rate less than their win learning rate. This significant *learning rate adjustment bias* was mainly driven by decreased loss learning rate adjustment but also by relatively increased win learning rate adjustment. Reduced adjustment of loss learning rates might lead individuals to experience negative events as less predictable and avoidable [17, 18]. The relative increase in win learning rates adjustment was unexpected. Our paradigm required simultaneous tracking of rewards and punishments. One potential explanation for the increase in win learning rate adjustment might be that participants with low mood had difficulties tracking the informativeness of losses and therefore focused their cognitive resources on tracking the informativeness of wins as a compensatory strategy.

Bifrontal tDCS applied during task performance normalised learning rate adjustment in low mood, both by increasing loss and decreasing win learning rate adjustment to changes in informativeness. This single-session experimental effect establishes proof-of-concept. If scaled up therapeutically, in principle, such an effect could potentially help individuals with anxious-depressive symptoms to make improved decisions in response to negative feedback and to overcome feelings of fear evoked by unpredictable negative outcomes [17]. Further research is needed to test whether this short-lasting tDCS effect can be prolonged with a training protocol and whether it transfers to untrained task contexts and generalizes to improve mood.

TDCS normalised learning rate adjustment bias only when applied *during*, but not *before* task performance (although their direct contrast was only a trend-level effect). Hence, to normalise behaviour, stimulation had to be applied during learning. This is consistent with our prediction, which arose from previous work, that tDCS must be paired during activity-dependent plasticity to modify learning [28, 36]. By contrast, in clinical trials, tDCS is usually applied at rest. We propose that tDCS may be more effective functionally and therapeutically if applied during a learning task that is relevant to depression instead of during rest.

A limitation of this study was the use of two different computational modelling approaches. The findings were observed only in the model in which the inverse temperature was kept constant across all blocks. It is unclear why the block-wise modelling approach used previously [19, 27, 37] yielded different results. Importantly, the key effect of tDCS (i.e. the increase in loss learning rate adjustment) was confirmed in the logistic regression analysis which was run as non-computational validation. Real compared to sham tDCS increased adjustment of the regression weight of the previous loss outcome to informativeness, which is conceptually equivalent to the increase in loss learning rate adjustment observed in the model. The effect on weight adjustment correlated with the effect on learning rate adjustment across participants, indicating that these two measures might capture similar behaviour. Therefore, both computational and non-computational analyses converged on the same conclusion that tDCS normalised negatively biased learning rate adjustment in low mood. Results from the regression analysis were less clear regarding the effect of low mood on learning rate adjustment. However, decreased loss learning rate adjustment has been reported previously in the literature [17, 18]. Replication studies are needed to assess the reliability of the findings observed here.

To conclude, this study found that low mood was associated with a specific deficit in adjusting learning to the volatility of loss outcomes. Bifrontal tDCS applied during (but not before) learning normalised this deficit. This experimental medicine study establishes proof-of-concept that tDCS can remediate learning deficits thought to underlie negative biases and impaired decision-making in depression. Future work will test whether this acute effect has therapeutic potential.

## Deviations from pre-registration

The analysis of the effect of low mood on learning rates was not pre-registered. We have added this analysis because it is useful for interpreting the effect of tDCS in low mood. The analysis suggests that in comparison to healthy individuals, individuals with low mood might have deficits in adjusting learning rates to volatility, and tDCS might normalise this deficit.

Our pre-registration did not include the ‘constant model’ approach (constant inverse temperature). We added this simpler modelling approach since the inverse temperature did not differ between task conditions in our previous work in healthy individuals [19]. In this study, the “constant model” approach captured predicted differences between healthy volunteers and low mood, which the “block-wise” approach was not able to detect.

We did not specify in our pre-registration how we would approach removal of potential outliers. We decided to repeat our analysis after removal of potential outliers more than 1.5 times the interquartile range below the first or above the third quartile, which is a common method for outlier identification [38, 39]. We have reported statistics for the complete dataset, both before and after outlier removal.

## Supplementary information

Supplementary Material

## Data Availability

Data and analysis scripts are available on Open Science Framework (10.17605/OSF.IO/KJB6Y).

## References

[1] Grimm S, Beck J, Schuepbach D, Hell D, Boesiger P, Bermpohl F, et al. Imbalance between left and right dorsolateral prefrontal cortex in major depression is linked to negative emotional judgment: an fMRI study in severe major depressive disorder. Biol Psychiatry. 2008;63:369–76. 17888408 10.1016/j.biopsych.2007.05.033

[2] Disner SG, Beevers CG, Haigh EA, Beck AT. Neural mechanisms of the cognitive model of depression. Nat Rev Neurosci. 2011;12:467–77. 21731066 10.1038/nrn3027

[3] Nitsche MA, Paulus W. Excitability changes induced in the human motor cortex by weak transcranial direct current stimulation. The Journal of physiology. 2000;527:633. 10990547 10.1111/j.1469-7793.2000.t01-1-00633.x PMC2270099

[4] Bergmann TO, Groppa S, Seeger M, Molle M, Marshall L, Siebner HR. Acute changes in motor cortical excitability during slow oscillatory and constant anodal transcranial direct current stimulation. J Neurophysiol. 2009;102:2303–11. 19692511 10.1152/jn.00437.2009

[5] Razza LB, Palumbo P, Moffa AH, Carvalho AF, Solmi M, Loo CK, et al. A systematic review and meta-analysis on the effects of transcranial direct current stimulation in depressive episodes. Depress Anxiety. 2020;37:594–608. 32101631 10.1002/da.23004

[6] Bradley BP, Mogg K, Williams R. Implicit and explicit memory for emotion-congruent information in clinical depression and anxiety. Behaviour research and therapy. 1995;33:755–70. 7677713 10.1016/0005-7967(95)00029-w

[7] Gotlib IH. Perception and recall of interpersonal feedback: Negative bias in depression. Cognitive Therapy and Research. 1983;7:399–412.

[8] Everaert J, Podina IR, Koster EHW. A comprehensive meta-analysis of interpretation biases in depression. Clin Psychol Rev. 2017;58:33–48. 28974339 10.1016/j.cpr.2017.09.005

[9] Godlewska BR, Browning M, Norbury R, Cowen PJ, Harmer CJ. Early changes in emotional processing as a marker of clinical response to SSRI treatment in depression. Transl Psychiatry. 2016;6:e957. 27874847 10.1038/tp.2016.130 PMC5314109

[10] Browning M, Kingslake J, Dourish CT, Goodwin GM, Harmer CJ, Dawson GR. Predicting treatment response to antidepressant medication using early changes in emotional processing. Eur Neuropsychopharmacol. 2019;29:66–75. 30473402 10.1016/j.euroneuro.2018.11.1102

[11] Godlewska BR, Norbury R, Selvaraj S, Cowen PJ, Harmer CJ. Short-term SSRI treatment normalises amygdala hyperactivity in depressed patients. Psychol Med. 2012;42:2609–17. 22716999 10.1017/S0033291712000591 PMC3488813

[12] Ironside M, O’Shea J, Cowen PJ, Harmer CJ. Frontal Cortex Stimulation Reduces Vigilance to Threat: Implications for the Treatment of Depression and Anxiety. Biol Psychiatry. 2016;79:823–30. 26210058 10.1016/j.biopsych.2015.06.012

[13] Ironside M, Browning M, Ansari TL, Harvey CJ, Sekyi-Djan MN, Bishop SJ, et al. Effect of Prefrontal Cortex Stimulation on Regulation of Amygdala Response to Threat in Individuals With Trait Anxiety: A Randomized Clinical Trial. JAMA Psychiatry. 2019;76:71–78. 30347011 10.1001/jamapsychiatry.2018.2172 PMC6583758

[14] Behrens TE, Woolrich MW, Walton ME, Rushworth MF. Learning the value of information in an uncertain world. Nat Neurosci. 2007;10:1214–21. 17676057 10.1038/nn1954

[15] Yu AJ, Dayan P. Uncertainty, neuromodulation, and attention. Neuron. 2005;46:681–92. 15944135 10.1016/j.neuron.2005.04.026

[16] Behrens TE, Hunt LT, Woolrich MW, Rushworth MF. Associative learning of social value. Nature. 2008;456:245–9. 19005555 10.1038/nature07538 PMC2605577

[17] Browning M, Behrens TE, Jocham G, O’Reilly JX, Bishop SJ. Anxious individuals have difficulty learning the causal statistics of aversive environments. Nat Neurosci. 2015;18:590–6. 25730669 10.1038/nn.3961 PMC4644067

[18] Gagne C, Zika O, Dayan P, Bishop SJ. Impaired adaptation of learning to contingency volatility in internalizing psychopathology. Elife. 2020;9:e61387. 33350387 10.7554/eLife.61387 PMC7755392

[19] Pulcu E, Browning M. Affective bias as a rational response to the statistics of rewards and punishments. Elife. 2017;6:e27879. 28976304 10.7554/eLife.27879 PMC5633345

[20] Pulcu E, Browning M. The Misestimation of Uncertainty in Affective Disorders. Trends Cogn Sci. 2019;23:865–75. 31431340 10.1016/j.tics.2019.07.007

[21] Cavanagh JF, Bismark AW, Frank MJ, Allen JJB. Multiple Dissociations Between Comorbid Depression and Anxiety on Reward and Punishment Processing: Evidence From Computationally Informed EEG. Comput Psychiatr. 2019;3:1–17. 31149639 10.1162/cpsy_a_00024 PMC6515849

[22] Aylward J, Valton V, Ahn WY, Bond RL, Dayan P, Roiser JP, et al. Altered learning under uncertainty in unmedicated mood and anxiety disorders. Nat Hum Behav. 2019;3:1116–23. 31209369 10.1038/s41562-019-0628-0 PMC6790140

[23] Pike AC, Robinson OJ. Reinforcement Learning in Patients With Mood and Anxiety Disorders vs Control Individuals: A Systematic Review and Meta-analysis. JAMA Psychiatry. 2022;79:313–2. 10.1001/jamapsychiatry.2022.0051 PMC8892374 35234834

[24] Massi B, Donahue CH, Lee D. Volatility Facilitates Value Updating in the Prefrontal Cortex. Neuron. 2018;99:598–608 e594. 30033151 10.1016/j.neuron.2018.06.033 PMC6085887

[25] Farashahi S, Donahue CH, Hayden BY, Lee D, Soltani A. Flexible combination of reward information across primates. Nat Hum Behav. 2019;3:1215–24. 31501543 10.1038/s41562-019-0714-3 PMC6856432

[26] Haber SN, Knutson B. The reward circuit: linking primate anatomy and human imaging. Neuropsychopharmacology. 2010;35:4–26. 19812543 10.1038/npp.2009.129 PMC3055449

[27] Overman MJ, Sarrazin V, Browning M, O’Shea J. Stimulating human prefrontal cortex increases reward learning. Neuroimage. 2023;271:120029. 36925089 10.1016/j.neuroimage.2023.120029 PMC11968408

[28] Fritsch B, Reis J, Martinowich K, Schambra HM, Ji Y, Cohen LG, et al. Direct current stimulation promotes BDNF-dependent synaptic plasticity: potential implications for motor learning. Neuron. 2010;66:198–204. 20434997 10.1016/j.neuron.2010.03.035 PMC2864780

[29] O’Shea J, Revol P, Cousijn H, Near J, Petitet P, Jacquin-Courtois S, et al. Induced sensorimotor cortex plasticity remediates chronic treatment-resistant visual neglect. Elife. 2017;6:e26602. 28893377 10.7554/eLife.26602 PMC5595432

[30] Li LM, Violante IR, Leech R, Ross E, Hampshire A, Opitz A, et al. Brain state and polarity dependent modulation of brain networks by transcranial direct current stimulation. Hum Brain Mapp. 2019;40:904–15. 30378206 10.1002/hbm.24420 PMC6387619

[31] Beck AT, Steer RA, Brown GK. Manual for the beck depression inventory-II. San Antonio, TX; Psychological Corporation: 1996.

[32] Stan Developement Team. Stan Modeling Language Users Guide and Reference Manual, version 2.30. 2022.

[33] Kunisato Y, Okamoto Y, Ueda K, Onoda K, Okada G, Yoshimura S, et al. Effects of depression on reward-based decision making and variability of action in probabilistic learning. J Behav Ther Exp Psychiatry. 2012;43:1088–94. 22721601 10.1016/j.jbtep.2012.05.007

[34] Mukherjee D, Filipowicz ALS, Vo K, Satterthwaite TD, Kable JW. Reward and punishment reversal-learning in major depressive disorder. J Abnorm Psychol. 2020;129:810–23. 33001663 10.1037/abn0000641

[35] Beevers CG, Worthy DA, Gorlick MA, Nix B, Chotibut T, Todd Maddox W. Influence of depression symptoms on history-independent reward and punishment processing. Psychiatry Res. 2013;207:53–60. 23122555 10.1016/j.psychres.2012.09.054 PMC3566413

[36] Reis J, Schambra HM, Cohen LG, Buch ER, Fritsch B, Zarahn E, et al. Noninvasive cortical stimulation enhances motor skill acquisition over multiple days through an effect on consolidation. Proc Natl Acad Sci USA. 2009;106:1590–5. 19164589 10.1073/pnas.0805413106 PMC2635787

[37] Pulcu E, Shkreli L, Holst CG, Woud ML, Craske MG, Browning M, et al. The Effects of the Angiotensin II Receptor Antagonist Losartan on Appetitive Versus Aversive Learning: A Randomized Controlled Trial. Biol Psychiatry. 2019;86:397–404. 31155138 10.1016/j.biopsych.2019.04.010

[38] Stevens J. Applied multivariate statistics for the social sciences. Mahwah, NJ: Lawrence Erlbaum Associates; 2022.

[39] Bakker M, Wicherts JM. Outlier removal, sum scores, and the inflation of the Type I error rate in independent samples t tests: the power of alternatives and recommendations. Psychol Methods. 2014;19:409–27. 24773354 10.1037/met0000014

[40] Laakso I, Tanaka S, Mikkonen M, Koyama S, Sadato N, Hirata A. Electric fields of motor and frontal tDCS in a standard brain space: A computer simulation study. Neuroimage. 2016;137:140–51. 27188218 10.1016/j.neuroimage.2016.05.032

